# Direct Ryanodine Receptor-2 Knockout in Primary Afferent Fibers Modestly Affects Neurological Recovery after Contusive Spinal Cord Injury

**DOI:** 10.1089/neur.2022.0044

**Published:** 2022-10-06

**Authors:** Ben C. Orem, Johnny R. Morehouse, Spencer Ames, Darlene A. Burke, David S.K. Magnuson, David P. Stirling

**Affiliations:** ^1^ Kentucky Spinal Cord Injury Research Center, University of Louisville, School of Medicine, Louisville, Kentucky, USA.; ^2^ Department of Neurological Surgery, University of Louisville, School of Medicine, Louisville, Kentucky, USA.; ^3^ Department of Anatomical Sciences and Neurobiology, University of Louisville, School of Medicine, Louisville, Kentucky, USA.; ^4^ Department of Microbiology and Immunology, University of Louisville, School of Medicine, Louisville, Kentucky, USA.

**Keywords:** axon degeneration, ryanodine receptor, secondary degeneration, spinal cord injury

## Abstract

Neuronal ryanodine receptors (RyR) release calcium from internal stores and play a key role in synaptic plasticity, learning, and memory. Dysregulation of RyR function contributes to neurodegeneration and negatively impacts neurological recovery after spinal cord injury (SCI). However, the individual role of RyR isoforms and the underlying mechanisms remain poorly understood. To determine whether RyR2 plays a direct role in axonal fate and functional recovery after SCI, we bred *Advillin*-Cre: tdTomato (Ai9) reporter mice with “floxed” RyR2 mice to directly knock out (KO) RyR2 function in dorsal root ganglion neurons and their spinal projections. Adult 6- to 8-week-old RyR2KO and littermate controls were subjected to a contusive SCI and their dorsal column axons were imaged *in vivo* using two-photon excitation microscopy. We found that direct RyR2KO in dorsal column primary afferents did not significantly alter secondary axonal degeneration after SCI. We next assessed behavioral recovery after SCI and found that direct RyR2KO in primary afferents worsened open-field locomotor scores (Basso Mouse Scale subscore) compared to littermate controls. However, both TreadScan™ gait analysis and overground kinematic gait analysis tests revealed subtle, but no fundamental, differences in gait patterns between the two groups after SCI. Subsequent removal of spared afferent fibers using a dorsal column crush revealed similar outcomes in both groups. Analysis of primary afferents at the lumbar (L3–L5) level similarly revealed no noticeable differences between groups. Together, our results support a modest contribution of dorsal column primary afferent RyR2 in neurological recovery after SCI.

## Introduction

Neurological recovery after spinal cord injury (SCI) is dependent on the location and extent of mechanical impact, delayed loss of spared tissue that succumbs to secondary degeneration, and neuroplasticity of remaining circuitry. Known mediators of secondary degeneration include vascular changes, neuroinflammation, edema, biochemical changes, and loss of ionic homeostasis.^1^ With regard to the latter, it is well known that pathological Ca^2+^ overload in white matter contributes to secondary degeneration; however, clinically viable and efficacious approaches to prevent Ca^2+^ overload remain elusive.^2^

Several extracellular routes of Ca^2+^ influx in axons have been implicated in secondary degeneration, including voltage-gated Ca^2+^ channels, reversal of Na^+^/Ca^2+^ exchange, ionotropic and metabotropic glutamate receptors, and mechanopore formation.^3–9^ Despite initial promising results, targeting many of the above “outside-in” sources of Ca^2+^ have proven to be ineffective in animal models of SCI^9–13^ and after a clinical trial.^14^

We and others have shown that “inside-out” sources of Ca^2+^, that is, those that reside within the axon, can also contribute to axonal injury.^10–13^ In support, the axoplasmic reticulum, the major Ca^2+^ store in axons, releases Ca^2+^ through ryanodine receptors (RyR) and IP_3_R,^11,13–15^ and both have been implicated in white matter injury.^10–13,15,16^ RyR are Ca^2+^ release channels that amplify cytosolic Ca^2+^ levels by releasing Ca^2+^ from intracellular Ca^2+^ stores and are expressed in most tissues, including the central nervous system (CNS).^17,18^ There are three known isoforms (RyR1, -2, and -3), and RyR1 and -2 mediate excitation-contraction coupling in skeletal and cardiac muscle, respectively.^19,20^ All three RyR isoforms are widely expressed in the CNS^21^ and are localized to neuronal soma, axons, dendrites, and spines^22–24^ and glial cells.^25–27^ Loss-of-function studies have implicated RyRs in synaptic plasticity, learning and memory, pain, and neurodegeneration.^28–33^

Globally targeting RyR has been shown to be axoprotective after traumatic^10,11,13^ and ischemic injury^12^ to spinal cord white matter *ex vivo* and after contusive SCI *in vivo*.^10^ RyR2 and RyR3 messenger RNA are elevated in dorsal root ganglions (DRGs) and at the lesion site, as well as localized to dystrophic axons and end-bulbs acutely after SCI.^34^ Further, RyR2 protein expression has been shown to increase at 1, 14, and 28 days at the lesion site post-SCI.^34^ Gain-of-function mutations of RyR2 worsen secondary axonal degeneration after SCI^15^ and systemic knockdown of RyR2 improves recovery after SCI, suggestive that RyR2 plays an important role in SCI pathophysiology. However, the relative contributions of each RyR isoform and whether they play a direct role in axonal pathology after SCI remain unknown.

To determine whether RyR2 plays a direct role in secondary axonal degeneration after SCI, we generated RyR2 knockout (KO) mice directly in sensory neurons by breeding “floxed” RyR2 KO mice with *Advillin-*Cre: tdTomato+ reporter mice (*Avil*-Cre:tdTom^fl/fl^:*Ryr2*^fl/fl^). Mice received a contusive SCI and intravital microscopy was used to observe dynamic changes in tdTomato^+^ axons in real time, and behavioral tests were performed to assess functional recovery. We report that direct RyR2 KO in DRG neurons did not prevent secondary axonal degeneration of the dorsal column projecting axons. Although RyR2 KO in primary afferents worsened functional recovery, as assessed using Basso Mouse Scale (BMS) subscores, detailed gait analysis using TreadScan™ and overground kinematics revealed that their overall gait patterns were not different.

## Methods

### Subjects

All experiments were approved by the University of Louisville Institutional Animal Care Committee, adhering to the Guide for the Care and Use of Laboratory Animals. Adult 6- to 8-week-old male and female Advillin (*Avil)*-Cre^+^:(Ai9) tdTom^fl/fl^:*Ryr2*^fl/fl^ and *Avil*-Cre^+^:tdTom^fl/fl^:*Ryr2*^wt/wt^ transgenic mice were established and bred in-house. The *Avil*-Cre^+^:tdTom^fl/fl^ reporter mouse line was obtained from Dr. Jeffrey C. Petruska, and the *Ryr2*^fl/fl^ mouse line was a kind gift from Dr. S.R. Wayne Chen. Both strains have been previously used and described in detail here.^35–37^ Because of the Advillin promoter driving Cre recombinase, tdTomato (strain #007909; The Jackson Laboratory, Bar Harbor, ME) is expressed and RyR2 knocked out only in Advillin-expressing cells. In the context of thoracic SCI, only DRG neurons that project their axons to and within the spinal cord will be tdTomato positive and lack functional RyR2. Genotyping confirmed the presence or absence of Cre, Ai9 (tdTomato), and Ryr2 floxed alleles in offspring.

A total of 32 adult 6-to 8-week-old male and female mice (16 Ryr2KO and 16 wild-type [WT] mice) were used in this study and equally divided between the *in vivo* imaging and behavioral experiments. Two RyR2 WT mice were excluded from the imaging study because of damage to the spinal cord during laminectomy and baseline imaging. Two RyR2 KO mice were excluded from the 7-day imaging sessions because of damage to the spinal cord while removing scar tissue that formed over the laminectomy site between the 1- and 7-day imaging sessions. Two mice were eliminated from the behavior study; 1 mouse did not recover from the contusive SCI surgery, and 1 mouse died before the 1-week behavioral testing, possibly because of bladder complications.

### Surgery

Mice were anesthetized with isoflurane and received saline and buprenorphine to manage pain. Body temperature was maintained at 37°C using a heating pad. Soothe^®^ lubricant eye ointment (Valeant Pharmaceuticals North America LLC, Bridgewater, NJ) was used to protect the eyes during surgery. A custom spinal cord clamp was used to firmly hold the vertebral column as previously described.^38^ A laminectomy was performed at T12 to allow for baseline recordings of tdTomato-positive axons within the superficial dorsal columns, and then mice received a 30-kdyn contusive SCI (Infinite Horizon Impactor Device, Precision Systems and Instrumentation LLC, Lexington, KY) with a dwell time of 0 sec. The location and mild injury for imaging experiments was chosen to limit breathing and motion artifacts and allow enough surviving dorsal column axons to assess secondary axonal degeneration respectively.^39^

Mice were then repositioned under the microscope to collect four-dimensional two-photon excitation (2PE) images at 1 and 7 days after injury. Post-surgery care included lactated Ringer's solution (2 cc, subcutaneous [s.c.]) given within 20 min after surgery, and gentamycin (Gentafuse; 5 mg/kg, s.c.) given within 20 min after surgery, and once-daily thereafter for 5 days to prevent infection. Mice also received buprenorphine once before surgery, and twice per day for 48 h to manage pain. After the 7-day image was collected, animals were euthanized, perfused with phosphate-buffered saline (PBS), and spinal cords fixed in 4% paraformaldehyde (PFA).

For the behavioral assessment experiments, mice were prepared as detailed above, but a 50-kdyn contusive SCI at T9 was used because the 30-kdyn force did not result in significant functional deficits between SCI animals and laminectomy-only controls (data not shown). After the sixth week of behavioral assessments, mice were anesthetized and subjected to a superficial dorsal column crush injury at T9 with Dumont number 5 forceps marked at a depth of 0.5 mm. Forceps were placed and squeezed and held together twice for a period of 10 sec each. Pre- and post-surgical care was identical as above. The purpose of the crush surgery was to eliminate the residual dorsal column projections at T9 without disrupting the underlying corticospinal tract and remove the former's contribution to spontaneous recovery after SCI. Behavioral tests on the dorsal column crush lesioned mice were carried out 2 weeks later as detailed below. After the last behavioral assessment, mice were euthanized, perfused with PBS followed by 4% PFA, and spinal cords fixed in 4% PFA overnight.

### Microscopy

An A1 × MP^+^ multi-photon confocal microscope (Nikon Instruments Inc., Melville, NY), equipped with a 25 × 1.1 numerical-aperture water immersion lens, was used for 2PE imaging of the spinal cord *in vivo* and images collected using Nikon Elements software. For the *in vivo* imaging experiments, the laser was tuned to 950 nm to excite tdTomato and 2PE images were collected at baseline and at the epicenter of the lesion site at 1 and 7 days after injury.

For assessment of post-mortem expression patterns of spinal tdTomato-positive fibers, confocal microscopy, using a 561-nm single-photon laser source, was used to capture tdTomato-positive afferent fiber expression patterns from transverse spinal cord sections that were collected at the lumbar level (L3–L5) of the spinal cord after the behavioral studies. These images were subsequently denoised to remove the shot-noise component from images using Denoise.ai on Nikon's Nis-Elements Advanced Research imaging software.

### Image analysis

All images were analyzed using the imaging processing software, ImageJ or Fuji (National Institutes of Health, Bethesda, MD), by experimenters' blind to genotype. For the *in vivo* imaging study, four-dimensional imaging data sets were collected, and axonal survival was quantified by drawing a perpendicular line (ImageJ; NIH) between the dorsal roots and dorsal vein at the epicenter of the lesion. All axons that touched the line were counted and divided by the length of the line and normalized to the pre-injury baseline axonal count. Axon survival was calculated as fractional survival. *N* = 6–8 per group was assessed, and mean ± standard deviation (SD) is reported (Fig. 2). Axonal spheroids were identified as axonal swellings with a diameter >3 times the mean axonal diameter at baseline. Retraction (End) bulbs were further identified if they had an axon connected only at one end. Spheroid and end-bulb counts were normalized to the visible area of the image to allow comparison between animals and quantified per mm^2^. *N* = 6–8 animals per group were assessed, and mean ± SD is reported (Fig. 2).

### Basso Mouse Scale and Basso Mouse Scale subscore

Hindlimb locomotor scores were assessed using the BMS, a 10-point scale ranging from 0 to 9 (no limb movement to normal locomotor function). Assessments were performed at pre-injury, 1 day, 3 days, and weekly post-injury for 6 weeks and then again 2 weeks post-dorsal crush by two trained observers who were blind to genotype. BMS subscores were also calculated when BMS hindlimb assessments reached a score of ≥5 as detailed here.^40^

### TreadScan gait analysis

Treadmill walking was assessed (Single Lane Gait Analysis Treadmill; Columbus Instruments, Columbus, OH) at pre-injury baseline, post-injury weekly for 6 weeks, and 2 weeks post-dorsal crush, as previously described.^41^ Briefly, as animals walked on the treadmill belt, a high-speed digital video camera recorded each pass from the ventral view. Digital video images were recorded at 100 frames/s and analyzed using TreadScan (Clever Sys Inc, Reston, VA). Acceptable passes included walking in the middle of the treadmill, stepping with few lateral deviations, and without pauses.

### Overground kinematics

Hindlimb kinematic analysis was performed as described previously.^42–44^ Animals walked freely inside a Plexiglass walkway (length × width × height: 155 × 10 × 24 cm) while high-speed (100 frames/s) videos were acquired from a ventral view using custom NI LabVIEW Software. Videos were then digitized using MaxTRAQ software (Innovision Systems, Columbiaville, MI), as previously described,^42,43^ by digitizing each paw as it was in contact with the ground for each pass. Coordinates from MaxTRAQ were then analyzed using a custom MS Excel add-in,^42,43^ which calculates all the gait measures. A minimum of three passes were analyzed that met the following criteria: continuous walking that started before the first step in the video; a relatively straight trajectory without hesitations; and was visually representative of animals' overall walking capabilities based on BMS scores.

### Horizontal ladder

The horizontal ladder test was performed pre-injury and biweekly (weeks 2, 4, and 6 post-injury and 2 weeks post-dorsal crush), using the Columbus Instruments Sensor and RS-232 Mini Counter (2.5 mm rungs spaced 3.5 cm apart; Columbus Instruments, Columbus, OH). Briefly, each animal underwent five stepping trials per session and an autocounter quantified the total number of footfalls. Only animals with a higher degree of weight-supported stepping ability were tested on the ladder post-injury (i.e., a bilateral BMS scores of at least 5 equaling frequent or consistent plantar stepping on both the left and right sides). Acceptable crossings could be obtained from either direction and were defined as those performed at a steady pace in which there was little or no hesitation or stopping and limbs were not dragged on the rungs. The five trials were averaged for each animal.

### Euthanasia, tissue processing, and image analysis

After the final imaging session and final behavior testing, mice were given pentobarbital for euthanasia and transcardially perfused with PBS, then 4% PFA. The entire brain and spinal cord were dissected and post-fixed in 4% PFA. Spinal cords were then transferred to 30% sucrose for cryoprotection for several days, and then the L1, L2, and L3–L5 segments were isolated, embedded in Tissue-Tek^®^ O.C.T. compound, and rapidly frozen on dry ice. Transverse 20-μm sections were collected on SuperFrost™ slides for assessment of primary afferent projections and terminations within the gray matter. Images of L3–L5 sections from both groups were imaged and visually compared (blind to treatment) for notable differences in termination patterns within the intermediate gray zone and ventral horns (*N* = 7 per group).

### Statistical analysis

Statistical analysis for all imaging experiments were performed using SigmaPlot software (Version 11; Systat Software Inc., San Jose, CA). We determined previously that an *n* = 6 per group was sufficient to determine a significant difference in secondary degeneration.^10,14^ For axonal survival, axonal spheroids, and end-bulbs, data are presented as mean ± SD, and group differences were assessed using a *t*-test. For the behavioral assessment groups, we determined that a group size of 8 was sufficient to achieve a desired power of 0.80, with a difference in means of 1.5, SD of 1.0, and alpha 0.05. Behavioral experimental data were analyzed using a repeated-measures analysis of variance (ANOVA) followed by Bonferroni's *post hoc t*-tests. Kinematic TreadScan and overground (except regularity index [RI] and coordinated pattern index [CPI]) outcome measures represent percentage of baseline values. Differences with a *p* value <0.05 were considered significant. Behavior assessments were analyzed using SPSS (v25; SPSS, Inc., Chicago, IL).

## Results

To determine a direct role for RyR2 in primary afferent secondary axonal degeneration and contributions to functional recovery after SCI, we generated a *Ryr2* loss-of-function transgenic mouse line. Mice bearing *Ryr*2 “floxed” alleles^35^ were crossed with mice expressing Cre recombinase under the *Advillin* promoter^37^ and floxed *Ai9* tdTomato reporter mouse line (*Avil-Cre:Ai9^fl/fl^*^)^). The resulting offspring were genotyped and *Avil*-Cre^+^:*Ai9^fl/fl^*:*Ryr2*^fl/fl^ KO mice and WT littermate controls (*Avil*-Cre^+^:*Ai9^fl/fl^*:*Ryr2*^wt/wt^) were used for all subsequent experiments. Thus, in the context of SCI, RyR2 KO is restricted to *Advillin*-expressing DRG neurons and their primary afferent axons, which terminate throughout the spinal cord gray matter and form the ascending dorsal columns.^37,45^

### Direct RyR2 knockout in dorsal column axons does not prevent axonal spheroid and end-bulb formation after contusive spinal cord injury

**FIG. 1.**
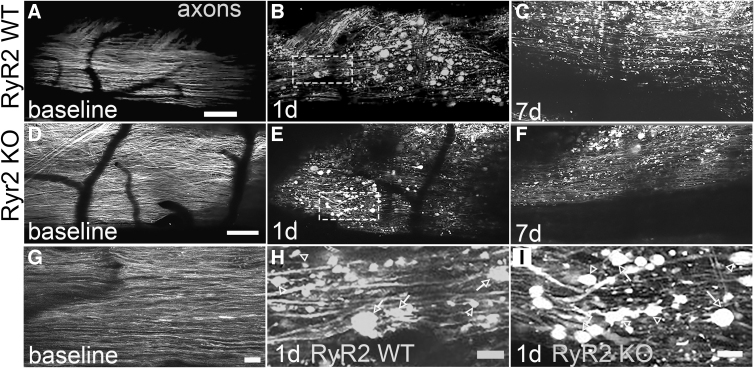
Effects of direct RyR2 KO in primary afferents on secondary axonal degeneration after contusive SCI. Representative maximum intensity projections of gracile fasciculus axons from *advil-Cre:tdtom^fl/fl^:Ryr2^wt/wt^* (referred to as RyR2WT; panels **A–C**) *and advil-Cre:tdtom^fl/fl^:Ryr2^fl/fl^* (referred to as RyR2KO; panels **D–F**) before and after a mild 30-kdyn T12 contusive SCI. All images were recorded *in vivo* using two-photon excitation microscopy. Axons (tdTomato+; gray) are shown in all images at baseline (**A,D,G**), 24 h (**B,E,H,I**), and 7 days after SCI (C,F). In baseline conditions, axons appear normal and linearly arranged with few signs of degeneration (A,D,G) regardless of genotype. In contrast, at 24 h after SCI, axonal spheroids (defined as axonal swellings with a diameter ≥3 times the mean axonal diameter at baseline; arrows) and end-bulbs (axonal swellings with diameters ≥3 times than their parent axon but had an axon connected at only one end; arrowheads) are present and represent the major axonal pathology at this time. By 7 days after injury, fewer axonal spheroids and end-bulbs are present regardless of genotype (C,F). Higher magnification images of axonal spheriods (arrows) and end-bulbs (arrowheads) are shown for RyR2WT mice (H; dashed rectangle region from panel B) and RyR2KO mice (I; dashed rectangle region from panel E), revealing very similar amounts of axonal degeneration between the groups. Scale bar: 100 μm in panels A–F; 20 μm in panels G–I. KO, knockout; SCI, spinal cord injury; WT, wild type.

As shown in Figure 1, *in vivo* imaging of superficial tdTomato^+^ dorsal column fibers revealed their linear arrangement without any apparent signs of axonal injury (i.e., axonal spheroids or end-bulbs) in baseline (laminectomy-only) conditions (Fig. 1A,D,G). In addition, there was no significant difference (*t*-test, *t* = −0.56, *df* = 12, *p* = 0.59) in the number of axonal spheroids between RyR2KO and WT littermate controls in baseline conditions (9.34 ± 12.51, mean ± SD; RyR2KO, *n* = 8 vs. 14.31 ± 18.92, RyR2WT, *n* = 6). Similarly, there was no significant difference (*t*-test, *t* = 0.15, *df* = 12, *p* = 0.89) in the number of axonal end-bulbs between groups in baseline conditions (2.67 ± 4.96, mean ± SD; RyR2KO, *n* = 8 vs. 2.32 ± 3.30, RyR2WT, *n* = 6). Importantly, these data reveal that the laminectomy to expose the spinal cord does not induce any overt damage to white matter.

**FIG. 2.**
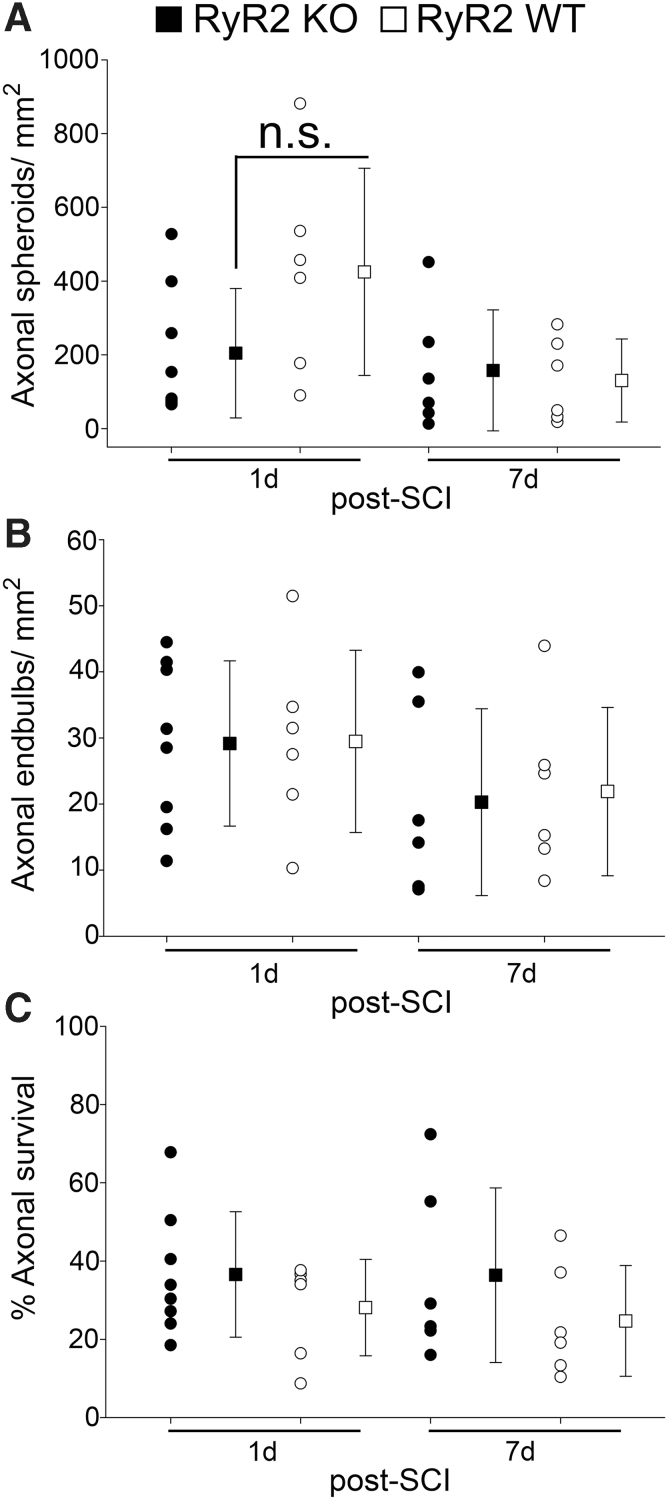
Direct RyR2 KO in primary afferents does not prevent axonal spheroid formation or end-bulb formation or increase axonal survival after contusive SCI. Quantification of axonal spheroid formation (**A**), End-bulb formation (**B**) and percent axonal survival (**C**) at 1 and 7 days after SCI reveal that there is no significant difference between RyR2KO (filled symbols) and RyR2WT (open symbols). Data are expressed as mean ± SD, and individual counts for each animal are also shown. *t*-test, *n* = 6–8 animals per group. Axonal spheroids: 1 day, *t* = −1.81, *df* = 12, *p* = 0.10; 7 days, *t* = 0.34, *df* = 10, *p* = 0.74. End-bulbs: 1 day, *t* = −0.05, *df* = 12, *p* = 0.964; 7 days, *t* = −0.21, *df* = 10, *p* = 0.84; percent axonal survival: 1 day, *t* = 1.08, *df* = 12, *p* = 0.30; 7 days, *t* = 1.08, *df* = 10, *p* = 0.30. KO, knockout; n.s., non-significant; SCI, spinal cord injury; SD, standard deviation; WT, wild type.

In contrast, at 1 day after a T12, 30-kdyn contusive SCI, many tdTomato-positive axons formed axonal spheroids and end-bulbs, resulting in significant loss of axons (Fig. 1B,E,H,I). By 7 days after SCI, fewer axonal spheroids and end-bulbs were evident in both RyR2KO and WT animals (Fig. 1C,F). Despite a trend for a reduction in acute axonal spheroid formation between RyR2 KO (204.48 ± 175.49, mean ± SD, *n* = 8) and RyR2 WT (425.05 ± 281.17, *n* = 6) controls, there was no significant difference (*t*-test, *t* = −1.81, *df* = 12, *p* = 0.10) in both axonal spheroid formation (Fig. 2A) and end-bulb formation (RyR2 KO; 29.16 ± 12.40, mean ± SD versus RyR2 WT; 29.48 ± 13.78, *t*-test, *t* = −0.046, *df* = 12, *p* = 0.96, *n* = 6–8 per group) at 1 day after SCI (Fig. 2B).

The same animals were then reimaged at 7 days post-SCI. Although, there were significantly less axonal spheroids (1 day RyR2 WT; 425.05 ± 281.18 vs. 7 days RyR2 WT; 130.57 ± 112.72, *t*-test, *t* = 2.38, *df* = 10, *p* = 0.039) at 7 days compared to 1 day after SCI, there was no significant difference between RyR2KO and RyR2WT controls (Figs. 1C,F and 2A). Similarly, there was no significant difference in end-bulb formation between groups at 7 days after SCI (Fig. 2B**)**.

### Direct RyR2 knockout in dorsal column axons does not prevent axonal loss after spinal cord injury

We next assessed axonal survival between RyR2 KO and RyR2 WT mice post-SCI. At both 24 h (RyR2 KO; percent axonal survival; 37.0 ± 16.0, mean ± SD vs. RyR2 WT; 28.1 ± 12.3, *t*-test, *t* = 1.08, *df* = 12, *p* = 0.30, *n* = 6–8 per group) and 7 days (RyR2 KO; 36.4 ± 22.3 vs. RyR2 WT; 24.7 ± 14.1, *t*-test, *t* = 1.08, *df* = 10, *p* = 0.30, *n* = 6 per group) after SCI, there was no significant difference in axonal survival between groups (Fig. 2C). There was also no significant difference between axonal loss for both groups of mice between 24 h and 7 days after SCI, suggestive that the spared tdTomato-positive axons within these groups remain stable during this period (Fig. 2C). Collectively, these data suggest that secondary degeneration of *advillin*-expressing DRG primary afferents post-SCI occurs in both the presence and absence of RyR2.

### *Direct RyR2 knockout in* Advillin*-expressing dorsal root ganglion spinal projections worsens open-field locomotor recovery after spinal cord injury*

The RyR2 isoform has been implicated in neuronal activity-induced Ca^2+^ release and synaptic plasticity^46,47^ and is localized to the soma, dendrites, spines, and axons.^34,47^ We hypothesized that loss of RyR2 function in primary afferents may alter sensory input required for normal coordinated stepping movements. To test this hypothesis, we assessed locomotor recovery of RyR2 KO and RyR2 WT mice in an open-field environment after a T12, 30-kdyn contusive SCI as used for the imaging studies. However, we were unable to detect clear functional deficits between contused and uninjured (laminectomy only) mice using standard BMS and BMS subscore assessments, possibly attributable to the mild nature of the injury (data not shown).

**FIG. 3.**
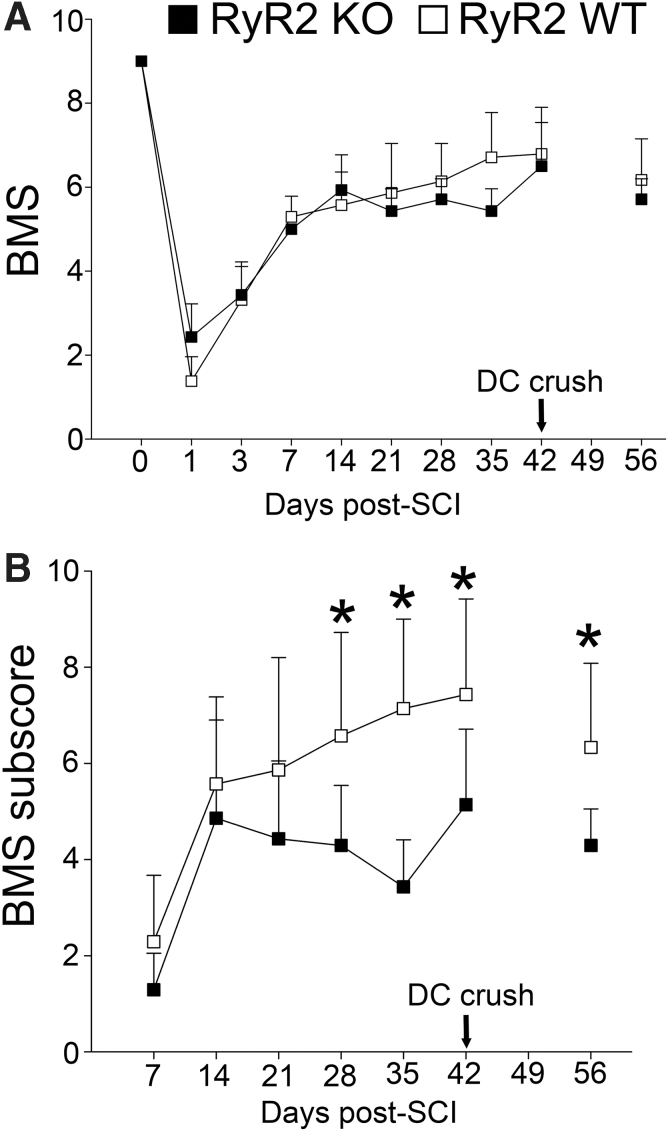
Direct RyR2 KO in primary afferents worsens open-field locomotor recovery after contusive SCI. Functional recovery was assessed after a T9, 50-kdyn IH contusion SCI for 42 days and then 14 days after a dorsal column (DC) crush injury using BMS (**A**) and BMS subscores (**B**). (A) BMS analysis of hindlimb locomotion revealed no significant group differences between RyR2KO and RyR2WT animals. However, the pattern of locomotor recovery is significantly different over time for the two groups. Data are expressed as mean ± SD. RM ANOVA (Group, *F* = 0.200, *df* = 1, 92, *p* = 0.20; Time, *F* = 5.80, *df* = 9, 23, *p* = 0.001; Group vs. Time, *F* = 2.6, *df* = 9, 23, *p* = 0.031; *n* = 7 per group). (B) BMS subscore analysis of hindlimb locomotion revealed a significant worsening of BMS subscores for RyR2KO versus RyR2WT animals from 28 to 42 days after a contusive SCI that also remained significant 14 days after DC crush. Data are expressed as mean ± SD. RM ANOVA (Group, *F* = 32.8, *df* = 1, 75, *p* < 0.001; Time, *F* = 18.9, *df* = 6, 23, *p* < 0.001; Group vs. Time, *F* = 1.6, *df* = 6, 23, *p* = 0.20; *n* = 7 per group). Group differences were determined using a Bonferroni *post hoc t*-test; *p < 0.05, **p < 0.01; *n* = 7 per group. BMS, Basso Mouse Scale; KO, knockout; RM ANOVA, repeated-measures analysis of variance; SCI, spinal cord injury; SD, standard deviation; WT, wild type.

We then subjected mice to a standard moderate T9/10, 50-kdyn contusive SCI that reproducibly induces hindlimb paralysis that spontaneously, but only partially, recovers after SCI.^8,40,48^ Although there were no significant group differences between BMS scores from baseline to 6 weeks after moderate SCI (Fig. 3A), the pattern of the locomotor recovery was different over time for the two groups (repeated-measures two-way ANOVA; group non-significant; Time, *p* < 0.001; Group by Time interaction, *p* < 0.05). However, when we tabulated BMS subscores that discriminate changes in fine details of locomotor recovery (stepping frequency, forelimb-hindlimb coordination, paw placement, and trunk stability), RyR2 KO mice had significantly worse recovery (*p* < 0.001) than RyR2 WT mice from 28 to 42 days (d28–42; *p* < 0.05) after contusive SCI (Fig. 3B). These data suggest that RyR2 expression in primary afferents may contribute to spontaneous functional recovery after SCI.

To test this hypothesis, we subjected these mice to a superficial (0.5-mm depth) dorsal column complete crush injury to determine whether RyR2 loss of function impacted residual and/or sprouting of primary afferents within the dorsal columns that may have contributed to the observed locomotor deficits between RyR2 KO and RyR2 WT mice. However, elimination of RyR2 function in these superficial dorsal column primary afferents resulted in similar BMS scores at 2 weeks after crush (i.e., 8 weeks after contusive SCI) compared to 6 weeks after contusive SCI (Fig. 3B). Interestingly, there remained a significant difference (*p* < 0.05, *t* = 2.82) in BMS subscores between RyR2KO (4.29 ± 0.76; mean ± SD) and RyR2WT (6.33 ± 1.75) groups at 2 weeks after a superficial dorsal column crush injury, which nearly mirrored the significant differences between groups at 6 weeks after contusion SCI. This suggests that the residual primary afferent fibers and other superficial fibers within the dorsal columns traversing the lesion site from either cohort were likely not involved in the observed functional differences between RyR2KO and RyR2WT animals.

### *Direct RyR2 KO in* Advillin*-expressing dorsal root ganglion spinal projections modestly affects gait after a contusive spinal cord injury*

**FIG. 4.**
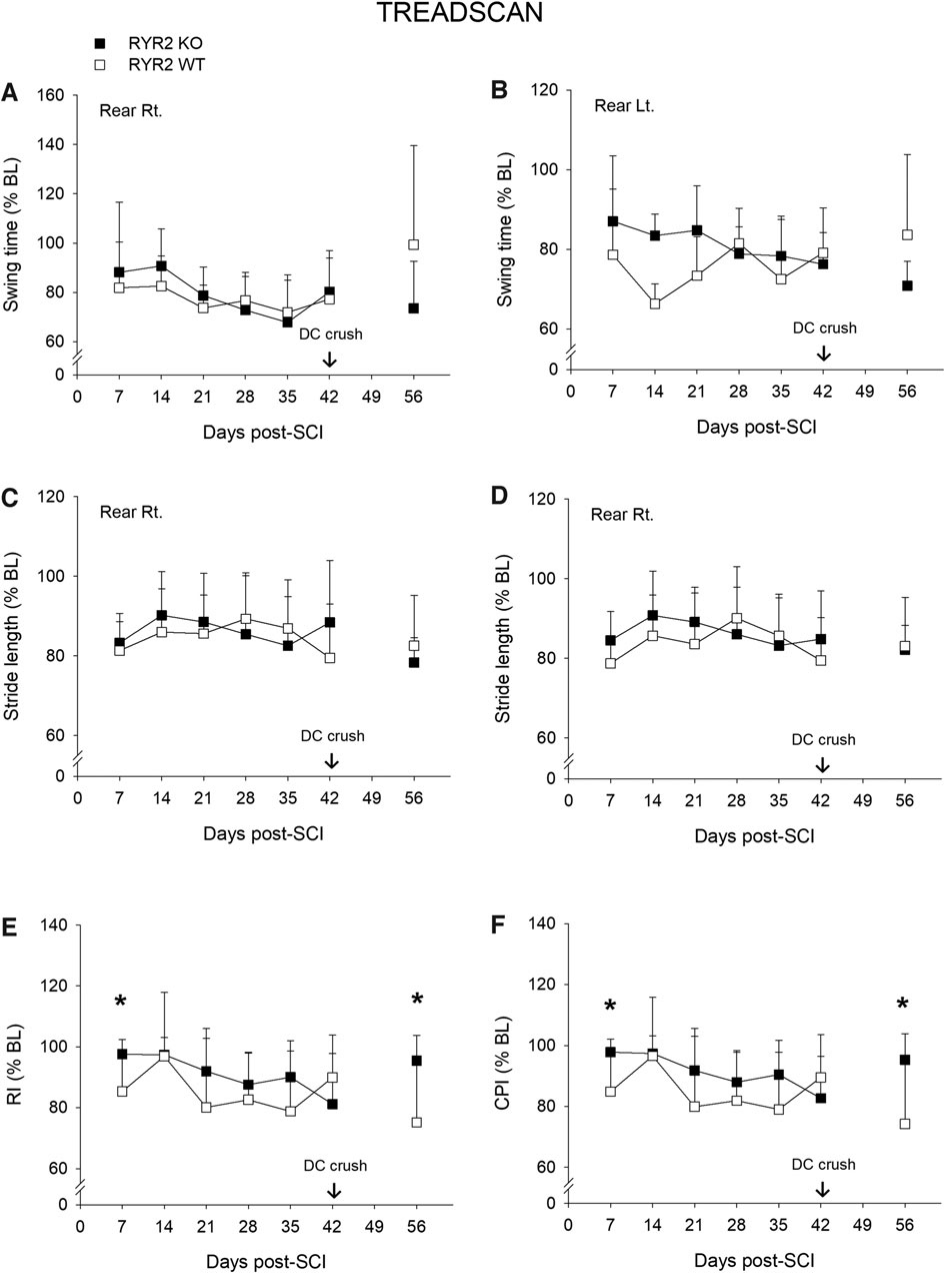
TreadScan analysis of gait after SCI reveals that direct RyR2 KO in primary afferents modestly affects coordinationed stepping. TreadScan assessment of gait parameters swing time (panel **A,** rear right [Rt.] paw; panel **B,** rear left [Lt.] paw) and stride length (panel **C,** rear Rt. paw; panel **D,** rear Lt. paw) reveals no significant group differences between RyR2KO and RyR2WT animals after contusive SCI and after a DC crush. Data are expressed as percent baseline; mean ± SD, *n* = 7 per group. TreadScan assessment of coordination using the regularity index (RI) (**E**) and coordinated pattern index (CPI) (**F**) shows that RyR2 KO significantly improved RI and CPI at 7 days after contusive SCI and again at 14 days after a DC crush injury. RI: RM ANOVA (*F* = 5.6, *df* = 1, 71, *p* = 0.020); Bonferroni's *post hoc t*-test, *p < 0.050. CPI: RM ANOVA (*F* = 7.1, *df* = 1, 69, *p* = 0.010); Bonferroni's *post hoc t*-test, *p < 0.050, *n* = 7 per group. DC, dorsal column; KO, knockout; RM ANOVA, repeated-measures analysis of variance; SCI, spinal cord injury; SD, standard deviation; WT, wild type.

**FIG. 5.**
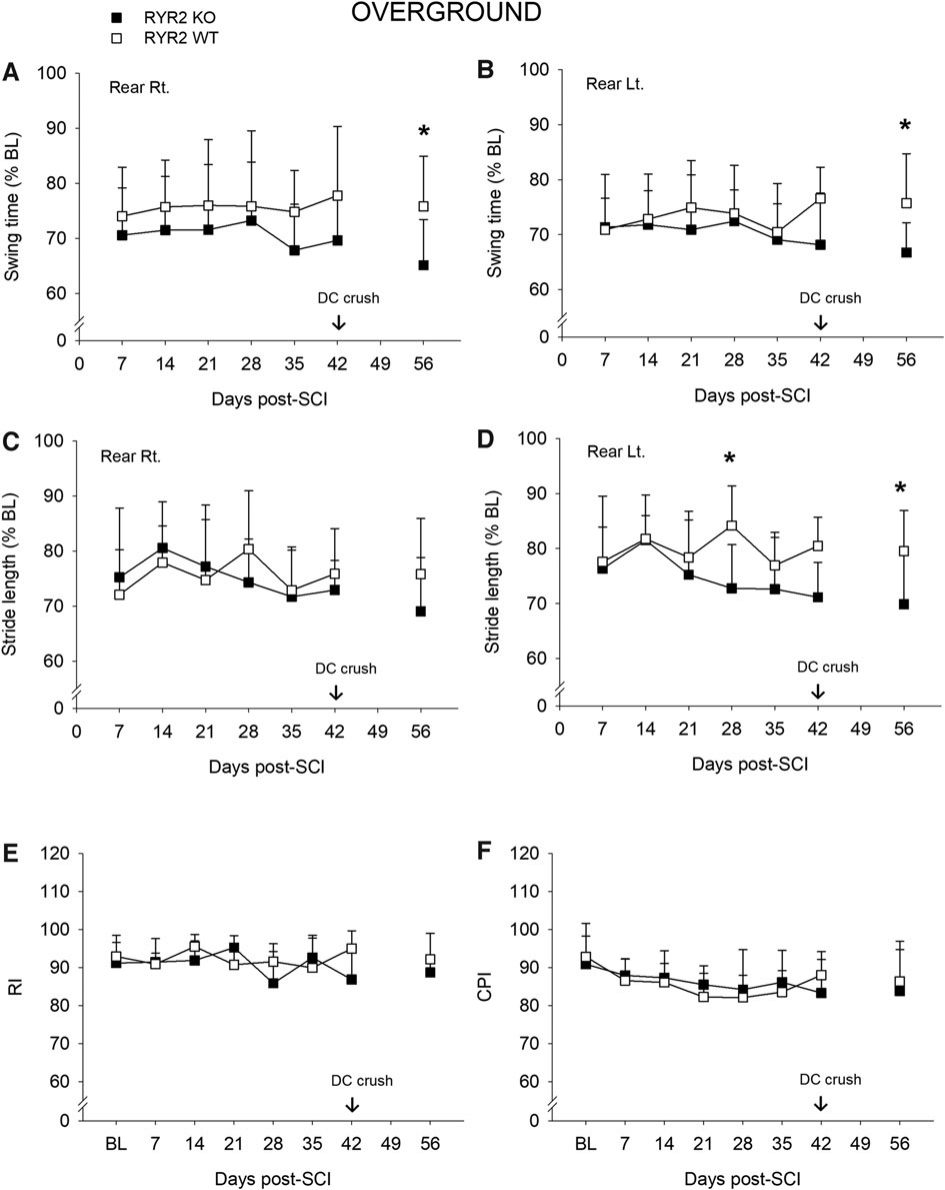
Overground kinematic analysis of gait after SCI reveals that direct RyR2 KO in primary afferents modestly alters swing time and stride length. Overground kinematic assessment of swing time reveals that there was no significant difference between groups up to 42 days after a contusive SCI. There was a significant difference between groups for swing time for the rear Rt. paw (panel **A**, RM ANOVA, *F* = 8.0, *df* = 1, 76, *p* = 0.010; Bonferroni's *post hoc t*-test, *p < 0.050) and rear Lt. Paw (panel **B**, *F* = 4.8, *df* = 1, 80, *p* = 0.032: Bonferroni's *post hoc t*-test, *p < 0.050) at 14 days after DC crush injury. Data are expressed as percent baseline; mean ± SD, *n* = 7 per group. (**C**) Rear Rt. stride length, N.S. (**D**) There was a significant difference for stride length between groups for the rear Lt. paw at 28 days post-contusive SCI and 14 days post-DC crush (both *p* < 0.050, Bonferroni's *post hoc t*-test). Data are expressed as percent baseline; mean ± SD, *n* = 7. There was no significant difference for both RI and CPI between RyR2KO and RyR2WT animals; mean ± SD, *n* = 7 per group. CPI, coordinated pattern index; DC, dorsal column; KO, knockout; N.S., non-significant; RI, regularity index; RM ANOVA, repeated-measures analysis of variance; SCI, spinal cord injury; SD, standard deviation; WT, wild type.

Concurrently with open-field observations, we performed more in-depth quantitative assessments of gait dynamics using TreadScan analysis, overground kinematics, and a horizonal ladder test. Overall, gait patterns were very similar between RyR2KO and their WT littermate controls for both swing time and stride length, with only modest differences observed at a single time point (compare TreadScan [Fig. 4A–D] and overground [Fig. 5A–D]). Interlimb coordination was also assessed using the RI and CPI. Both calculate the percentage of normal step-sequence patterns out of the total number of paw placements; however, the latter includes both dorsal and plantar foot placements. Though we did observe significant differences using TreadScan analysis between the RyR2KO and RyR2WT groups with indices of limb coordination, that is, the RI (*p* < 0.05) and CPI (*p* < 0.05), at 7 days after contusive SCI, the groups recovered similarly from 2 to 6 weeks after SCI (Fig. 4E,F). These differences in interlimb coordination were not supported using overground kinematic analysis (Fig. 5E,F). Further, we did not detect any significant differences between groups using the horizontal ladder test (42 days, RyR2KO 3.8 ± 3.3, mean ± SD footfalls vs. RyR2WT 2.6 ± 0.9, *F* = 1.8, *df* = 1,16, *p* = 0.20). Similarly, although we observed some significant differences between groups after a dorsal column crush injury, these differences were scattered across different measures and unsupported by other related outcomes, for example, TreadScan RI and CPI versus overground RI and CPI (compare Figs. 4E,F and 5E,F). Taken together, these data do not support a functionally significant role for primary afferent expression of RyR2 in gait recovery after SCI.

### *Direct RyR2 knockout in* Advillin*-expressing dorsal root ganglion neurons does not alter spinal projections and termination patterns within the L3–L5 level of the spinal cord*

**FIG. 6.**
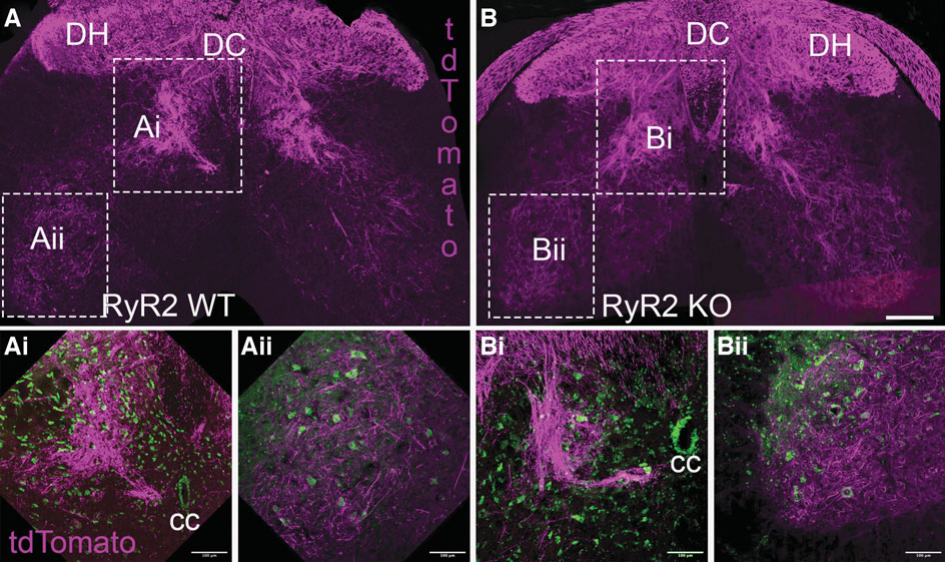
Direct RyR2 KO in DRG primary afferents does not notably alter their projection and termination patterns within the L3–L5 spinal cord gray matter. (**A,B**) Representative 10 × confocal images of transverse spinal cord sections from the L3–L5 level of the spinal cord, revealing the projection and termination patterns of primary afferent fibers (tdTomato+; magenta) from RyR2WT (**A,Ai,Aii**) and RyR2KO (**B,Bi,Bii**) animals post-SCI. As expected and based on their known role in sensation, the majority of DRG primary afferents are clearly visible within the dorsal columns (DC) and their terminations within the dorsal horn (DH) with a tapering of terminations within the intermediate gray region and ventral horns. Higher magnification images of the intermediate gray region (Ai,Bi) and ventral horn (Aii,Bii) are indicated in hashed boxes in panels A and B and were captured using confocal microscopy with a water-dipped 20 × objective. Termination patterns between groups were very similar at each location assessed (*n* = 7 per group). Primary afferent fibers (tdTomato; magenta; autofluoresence; green to indicate cell bodies). Scale bar: 100 μm. CC, central canal; DRG, dorsal root ganglion; KO, knockout; SCI, spinal cord injury; WT, wild type.

To compliment the behavioral studies, we assessed tdTomato^+^ primary afferent axons and their terminations in intermediate and ventral gray matter at the level of the L3–L5 spinal cord in RyR2WT and RyR2KO animals. As expected, the majority of tdTomato^+^ afferent axons were present within the ascending dorsal columns and absent within the underlying cortical spinal tract (Fig. 6). Most of these axonal projections terminated in the superficial layers of the gray matter (i.e., lamina I and II), with a decreasing number of projections and terminations in the deeper lamina. In agreement with the gait analysis, there were no notable differences in termination patterns between groups (Fig. 6A,B).

## Discussion

Collectively, our results do not implicate RyR2-mediated intra-axonal Ca^2+^ release in primary afferent fibers as an important contributor to delayed primary afferent degenerative phenomena after contusive SCI. In support, axonal spheroid and end-bulb formation and axonal survival were similar between both groups from 1 to 7 days after SCI. Despite a significant worsening of BMS subscores in RyR2 KO mice versus RyR2 WT controls, both TreadScan and overground kinematic analyses failed to detect a pattern of meaningful, robust, and lasting gait differences between groups. These data suggest that RyR2 expression in primary afferents does not play a major role in spontaneous recovery of gait after SCI.

There are several possibilities underlying why, in the current study, we failed to show an axoprotective effect by knocking out RyR2 directly in axons *in vivo*. Among the possibilities are: 1) Other RyR isoforms (i.e., RyR1 and/or -3) may play a more important role than RyR2 in axonal injury; 2) compensation through other RyR isoforms and/or IP_3_R may have masked the effect of RyR2 KO; 3) other routes of Ca^2+^ release and/or entry may play a larger role; and 4) indirect effects of RyR2 inhibition.

Given that previous studies have shown that targeting all RyR isoforms with ryanodine and other RyR antagonists are protective across a broad spectrum of white matter injury models (see Introduction), we cannot rule out the possibility that other RyR isoforms contributed to axonal loss. In addition, given that targeting other inside-out sources of Ca^2+^ (e.g., IP_3_R^9,14,49^ as well as mitochondria release of Ca^2+^),^49^ protects white matter, these additional sources could have played a role. As detailed in the Introduction, several sources of outside-in Ca^2+^ are well known to contribute to white matter injury that, together with inside-out sources, may lead to toxic levels of intra-axonal Ca^2+^ accumulation. Given the complexity and overlap of Ca^2+^ sources that are involved in white matter injury, further studies using combinatorial targeting strategies will likely be necessary to rescue white matter from secondary degeneration.

To the best of our knowledge, this is the first study to directly knock out an RyR in neurons and their spinal projections. We therefore cannot exclude the possibility that indirect effects of RyR inhibition, rather than direct effects on axons *per se*, may underlie the protective effects of RyR inhibition reported in previous studies. In support of this, most of the previous studies used *ex vivo* models of white matter injury that would also include additional RyR-expressing cells such as astrocytes, oligodendrocytes, and microglia. Further, the *in vivo* ischemic and trauma models would recruit additional blood-derived elements, including neutrophils and macrophages, that all express RyR receptors and are known to contribute to white matter injury. In support of this, Qian and colleagues showed that knockdown of RyR2 significantly improved locomotor recovery and reduced lesion size after SCI.^50^ Mechanistically, the RyR2 knockdown reduced proinflammatory cytokine levels, improved spinal cord oxygen consumption rate, reduced endoplasm reticulum stress, and oxidative stress through NADPH oxidase-2.^50^ Further studies using cell-type–specific KO of the different RyR isoforms will likely further our understanding of the complex role of RyR in CNS trauma and disease.

Our in-depth analysis of both treadmill (TreadScan) and overground locomotor characteristics failed to identify a meaningful pattern of differences in stepping parameters post-injury despite an initial observation of differences in BMS subscores for reasons unknown. Although it appears that RyR2 KO induced some subtle functional changes, overall, it is difficult to ascribe these changes to the initial rationale for the study. One potential explanation is that RyR2 KO involved an unrecognized sensory phenotype that, in turn, exercised a subtle impact on stepping that would require a much deeper investigation to uncover. This concept is supported by previous work implicating RyR2 in pain syndromes^30,51^; however, because we did not assess pain and nociception in the current study, this remains speculation.

Another potential limitation of our study was the use of a non-inducible transgenic mouse line that may unavoidably have acquired developmental and/or compensatory mechanisms of neuron-specific RyR2 KO beyond our control. Even though the *Advil*-Cre driver mouse line has been previously assessed and no developmental or behavioral deficits were evident,^37^ we detected minor, but significant, differences in gait at baseline between groups of mice. Although we normalized post-SCI outcomes to baseline, it is possible that the observed differences in gait detected post-SCI may reflect inherent underlying differences at baseline between RyR2WT and RyR2KO mice. Further work using an inducible-*Advil-*Cre may help avoid potential confounding developmental/compensatory effects. Last, we did not evaluate possible differences in synaptic function between WT and RyR2 KO animals that may have contributed to the subtle differences in gait. Collectively, this leads us to conclude that there were neither neuroprotective effects on axons nor real functionally significant outcomes attributable to direct RyR2KO in DRG primary afferents.

## Abbreviations Used

2PE: two-photon excitation
ANOVA: analysis of variance
BMS: Basso Mouse Scale
CNS: central nervous system
CPI: coordinated pattern index
DRG: dorsal root ganglion
KO: knockout
PBS: phosphate-buffered saline
PFA: paraformaldehyde
RI: regularity index
RyR: ryanodine receptors
x.c.: subcutaneous
SCI: spinal cord injury
SD: standard deviation
WT: wild type

## References

[1] Hachem LD, Fehlings MG. Pathophysiology of spinal cord injury. Neurosurg Clin N Am 2021;32(3):305–313; doi: 10.1016/j.nec.2021.03.002 34053718

[2] Baroncini A, Maffulli N, Eschweiler J, et al. Pharmacological management of secondary spinal cord injury. Expert Opin Pharmacother 2021;22(13):1793–1800; doi: 10.1080/14656566.2021.1918674 33899630

[3] O'Hare Doig RL, Santhakumar S, Fehily B, et al. Acute cellular and functional changes with a combinatorial treatment of ion channel inhibitors following spinal cord injury. Front Mol Neurosci 2020;13:85; doi: 10.3389/fnmol.2020.00085 PMC7331598 32670018

[4] Agrawal SK, Nashmi R, Fehlings MG. Role of L- and N-type calcium channels in the pathophysiology of traumatic spinal cord white matter injury. Neuroscience 2000;99(1):179–188; doi: 10.1016/s0306-4522(00)00165-2 10924962

[5] Imaizumi T, Kocsis JD, Waxman SG. The role of voltage-gated Ca^2+^ channels in anoxic injury of spinal cord white matter. Brain Res 1999;817(1–2):84–92; doi: 10.1016/s0006-8993(98)01214-1 9889329

[6] Stys PK. White matter injury mechanisms. Curr Mol Med 2004;4(2):113–130; doi: 10.2174/1566524043479220 15032708

[7] Shahsavani N, Kataria H, Karimi-Abdolrezaee S. Mechanisms and repair strategies for white matter degeneration in CNS injury and diseases. Biochim Biophys Acta Mol Basis Dis 2021;1867(6):166117; doi: 10.1016/j.bbadis.2021.166117 33667627

[8] Maxwell WL, Kobeissy FH, eds. Development of Concepts in the Pathology of Traumatic Axonal and Traumatic Brain Injury. In: Brain Neurotrauma: Molecular, Neuropsychological, and Rehabilitation Aspects. CRC Press/Taylor & Francis; Boca Raton, FL; 2015; Chapter 3.

[9] Stirling DP, Stys PK. Mechanisms of axonal injury: internodal nanocomplexes and calcium deregulation. Trends Mol Med 2010;16(4):160–170; doi: 10.1016/j.molmed.2010.02.002 20207196 PMC2976657

[10] Orem BC, Rajaee A, Stirling DP. Inhibiting calcium release from ryanodine receptors protects axons after spinal cord injury. J Neurotrauma 2022;39(3–4):311–319; doi: 10.1089/neu.2021.0350 34913747 PMC8817717

[11] Orem BC, Pelisch N, Williams J, et al. Intracellular calcium release through IP3R or RyR contributes to secondary axonal degeneration. Neurobiol Dis 2017;106:235–243; doi: 10.1016/j.nbd.2017.07.011 28709993

[12] Ouardouz M, Nikolaeva MA, Coderre E, et al. Depolarization-induced Ca^2+^ release in ischemic spinal cord white matter involves L-type Ca^2+^ channel activation of ryanodine receptors. Neuron 2003;40(1):53–63; doi: 10.1016/j.neuron.2003.08.016 14527433

[13] Thorell WE, Leibrock LG, Agrawal SK. Role of RyRs and IP3 receptors after traumatic injury to spinal cord white matter. J Neurotrauma 2002;19(3):335–342; doi: 10.1089/089771502753594909 11939501

[14] Orem BC, Rajaee A, Stirling DP. IP3R-mediated intra-axonal Ca(2+) release contributes to secondary axonal degeneration following contusive spinal cord injury. Neurobiol Dis 2020;146:105123; doi: 10.1016/j.nbd.2020.105123 PMC7686917 33011333

[15] Stirling DP, Cummins K, Wayne Chen SR, et al. Axoplasmic reticulum Ca(2+) release causes secondary degeneration of spinal axons. Ann Neurol 2014;75(2):220–229; doi: 10.1002/ana.24099 24395428

[16] Ouardouz M, Malek S, Coderre E, et al. Complex interplay between glutamate receptors and intracellular Ca^2+^ stores during ischaemia in rat spinal cord white matter. J Physiol 2006;577(Pt 1):191-204; doi: 10.1113/jphysiol.2006.116798 PMC2000677 16945971

[17] Santulli G, Lewis D, des Georges A, et al. Ryanodine receptor structure and function in health and disease. Subcell Biochem 2018;87:329–352; doi: 10.1007/978-981-10-7757-9_11 29464565 PMC5936639

[18] Dulhunty AF, Beard NA, Casarotto MG. Recent advances in understanding the ryanodine receptor calcium release channels and their role in calcium signalling. F1000Res 2018;7:F1000 Faculty Rev-1851; doi: 10.12688/f1000research.16434.1 PMC6259491 30542613

[19] Fill M, Ma JJ, Knudson CM, et al.. Role of the ryanodine receptor of skeletal muscle in excitation-contraction coupling. Ann N Y Acad Sci 1989;560:155–162; doi: 10.1111/j.1749-6632.1989.tb24092.x 2662857

[20] Eisner DA, Caldwell JL, Kistamas K, et al. Calcium and excitation-contraction coupling in the heart. Circ Res 2017;121(2):181–195; doi: 10.1161/CIRCRESAHA.117.310230 28684623 PMC5497788

[21] Sharp AH, McPherson PS, Dawson TM, et al. Differential immunohistochemical localization of inositol 1,4,5-trisphosphate- and ryanodine-sensitive Ca^2+^ release channels in rat brain. J Neurosci 1993;13(7):3051–3063; doi: 10.1523/JNEUROSCI.13-07-03051.1993 8392539 PMC6576698

[22] Sah P, Francis K, McLachlan EM, et al. Distribution of ryanodine receptor-like immunoreactivity in mammalian central nervous system is consistent with its role in calcium-induced calcium release. Neuroscience 1993;54(1):157–165; doi: 10.1016/0306-4522(93)90391-r 8390624

[23] Breit M, Kessler M, Stepniewski M, et al. Spine-to-dendrite calcium modeling discloses relevance for precise positioning of ryanodine receptor-containing spine endoplasmic reticulum. Sci Rep 2018;8(1):15624; doi: 10.1038/s41598-018-33343-9 PMC6199256 30353066

[24] Berridge MJ. Neuronal calcium signaling. Neuron 1998;21(1):13–26; doi: 10.1016/s0896-6273(00)80510-3 9697848

[25] Simpson PB, Holtzclaw LA, Langley DB, et al. Characterization of ryanodine receptors in oligodendrocytes, type 2 astrocytes, and O-2A progenitors. J Neurosci Res 1998;52(4):468–482; doi: 10.1002/(SICI)1097-4547(19980515)52:4<468::AID-JNR11>3.0.CO;2-# 9589392

[26] Matyash M, Matyash V, Nolte C, et al. Requirement of functional ryanodine receptor type 3 for astrocyte migration. FASEB J 2002;16(1):84–86; doi: 10.1096/fj.01-0380fje 11709492

[27] Klegeris A, Choi HB, McLarnon JG, et al. Functional ryanodine receptors are expressed by human microglia and THP-1 cells: their possible involvement in modulation of neurotoxicity. J Neurosci Res 2007;85(10):2207–2215; doi: 10.1002/jnr.21361 17526017

[28] Futagi D, Kitano K. Ryanodine-receptor-driven intracellular calcium dynamics underlying spatial association of synaptic plasticity. J Comput Neurosci 2015;39(3):329–347; doi: 10.1007/s10827-015-0579-z 26497496 PMC4648987

[29] Ferrari LF, Khomula EV, Araldi D, et al. Marked sexual dimorphism in the role of the ryanodine receptor in a model of pain chronification in the rat. Sci Rep 2016;6:31221; doi: 10.1038/srep31221 PMC4976309 27499186

[30] Godai K, Takahashi K, Kashiwagi Y, et al. Ryanodine receptor to mitochondrial reactive oxygen species pathway plays an important role in chronic human immunodeficiency virus gp120MN-induced neuropathic pain in rats. Anesth Analg 2019;129(1):276–286; doi: 10.1213/ANE.0000000000003916 30507840

[31] Berridge MJ. Calcium hypothesis of Alzheimer's disease. Pflugers Arch 2010;459(3):441–449; doi: 10.1007/s00424-009-0736-1 19795132

[32] More JY, Bruna BA, Lobos PE, et al. Calcium release mediated by redox-sensitive RyR2 channels has a central role in hippocampal structural plasticity and spatial memory. Antioxid Redox Signal 2018;29(12):1125–1146; doi: 10.1089/ars.2017.7277 29357673

[33] Johenning FW, Theis AK, Pannasch U, et al. Ryanodine receptor activation induces long-term plasticity of spine calcium dynamics. PLoS Biol 2015;13(6):e1002181; doi: 10.1371/journal.pbio.1002181 PMC4476683 26098891

[34] Pelisch N, Gomes C, Nally JM, et al. Differential expression of ryanodine receptor isoforms after spinal cord injury. Neurosci Lett 2017;660:51–56; doi: 10.1016/j.neulet.2017.09.018 28899787

[35] Bround MJ, Asghari P, Wambolt RB, et al. Cardiac ryanodine receptors control heart rate and rhythmicity in adult mice. Cardiovasc Res 2012;96(3):372–380; doi: 10.1093/cvr/cvs260 22869620 PMC3500041

[36] Orem BC, Partain SB, Stirling DP. Inhibiting store-operated calcium entry attenuates white matter secondary degeneration following SCI. Neurobiol Dis 2020;136:104718; doi: 10.1016/j.nbd.2019.104718 31846736

[37] Zurborg S, Piszczek A, Martinez C, et al. Generation and characterization of an Advillin-Cre driver mouse line. Mol Pain 2011;7:66; doi: 10.1186/1744-8069-7-66 PMC3185264 21906401

[38] Stirling DP, Liu S, Kubes P, et al. Depletion of Ly6G/Gr-1 leukocytes after spinal cord injury in mice alters wound healing and worsens neurological outcome. J Neurosci 2009;29(3):753–764; doi: 10.1523/JNEUROSCI.4918-08.2009 19158301 PMC6665178

[39] Rajaee A, Geisen ME, Sellers AK, et al. Repeat intravital imaging of the murine spinal cord reveals degenerative and reparative responses of spinal axons in real-time following a contusive SCI. Exp Neurol 2020;327:113258; doi: 10.1016/j.expneurol.2020.113258 PMC7549695 32105708

[40] Basso DM, Fisher LC, Anderson AJ, et al. Basso Mouse Scale for locomotion detects differences in recovery after spinal cord injury in five common mouse strains. J Neurotrauma 2006;23(5):635–659; doi: 10.1089/neu.2006.23.635 16689667

[41] Beare JE, Morehouse JR, DeVries WH, et al. Gait analysis in normal and spinal contused mice using the TreadScan system. J Neurotrauma 2009;26(11):2045–2056; doi: 10.1089/neu.2009.0914 19886808 PMC2813489

[42] Pocratsky AM, Shepard CT, Morehouse JR, et al. Long ascending propriospinal neurons provide flexible, context-specific control of interlimb coordination. Elife 2020 Sep 9;9:e53565; doi: 10.7554/eLife.53565 PMC7527236 32902379

[43] Pocratsky AM, Burke DA, Morehouse JR, et al. Reversible silencing of lumbar spinal interneurons unmasks a task-specific network for securing hindlimb alternation. Nat Commun 2017;8(1):1963; doi: 10.1038/s41467-017-02033-x PMC5719045 29213073

[44] Kuerzi J, Brown EH, Shum-Siu A, et al. Task-specificity vs. ceiling effect: step-training in shallow water after spinal cord injury. Exp Neurol 2010;224(1):178–187; doi: 10.1016/j.expneurol.2010.03.008 20302862 PMC2885471

[45] Hasegawa H, Abbott S, Han BX, et al. Analyzing somatosensory axon projections with the sensory neuron-specific Advillin gene. J Neurosci 2007;27(52):14404–14414; doi: 10.1523/JNEUROSCI.4908-07.2007 18160648 PMC6673440

[46] Lobos P, Cordova A, Vega-Vasquez I, et al. RyR-mediated Ca(2+) release elicited by neuronal activity induces nuclear Ca(2+) signals, CREB phosphorylation, and Npas4/RyR2 expression. Proc Natl Acad Sci U S A 2021;118(33):e2102265118; doi: 10.1073/pnas.2102265118 PMC8379958 34389673

[47] Shimizu H, Fukaya M, Yamasaki M, et al. Use-dependent amplification of presynaptic Ca^2+^ signaling by axonal ryanodine receptors at the hippocampal mossy fiber synapse. Proc Natl Acad Sci U S A 2008;105(33):11998–12003; doi: 10.1073/pnas.0802175105 18687898 PMC2575294

[48] Nishi RA, Liu H, Chu Y, et al. Behavioral, histological, and ex vivo magnetic resonance imaging assessment of graded contusion spinal cord injury in mice. J Neurotrauma 2007;24(4):674–689; doi: 10.1089/neu.2006.0204 17439350

[49] Villegas R, Martinez NW, Lillo J, et al. Calcium release from intra-axonal endoplasmic reticulum leads to axon degeneration through mitochondrial dysfunction. J Neurosci 2014;34(21):7179–7189; doi: 10.1523/JNEUROSCI.4784-13.2014 24849352 PMC4028495

[50] Liao B, Zhang Y, Sun H, et al. Ryanodine receptor 2 plays a critical role in spinal cord injury via induction of oxidative stress. Cell Physiol Biochem 2016;38(3):1129–1137; doi: 10.1159/000443063 26963898

[51] Sanna MD, Peroni D, Quattrone A, et al. Spinal RyR2 pathway regulated by the RNA-binding protein HuD induces pain hypersensitivity in antiretroviral neuropathy. Exp Neurol 2015;267:53–63; doi: 10.1016/j.expneurol.2015.02.036 25765490

